# GDF15 secreted by senescent endothelial cells improves vascular progenitor cell functions

**DOI:** 10.1371/journal.pone.0216602

**Published:** 2019-05-10

**Authors:** Guillaume Ha, Fanny De Torres, Nassim Arouche, Nassima Benzoubir, Ségolène Ferratge, Elie Hatem, Adrienne Anginot, Georges Uzan

**Affiliations:** 1 INSERM U1197, Hôpital Paul Brousse, Villejuif, France; 2 Université Paris-Diderot, Paris, France; 3 UMRS-1193, Hôpital Paul Brousse, Villejuif, France; 4 Cellenion, Hôpital Paul Brousse, Villejuif, France; Medical University Innsbruck, AUSTRIA

## Abstract

Endothelial dysfunction (ED) is part of the first steps in the development of cardiovascular diseases (CVD). Growth Differentiation Factor 15 (GDF15) is a cytokine belonging to the Transforming Growth Factor β superfamily and its expression is increased both during ED and in CVD. Because high blood levels of GDF15 have been reported during ED, we hypothesized that GDF15 could be produced by endothelial cells in response to a vascular stress, possibly to attenuate endothelial function loss. Since senescence is mainly involved in both vascular stress and endothelial function loss, we used Endothelial Colony Forming Cells generated from adult blood (AB-ECFCs) as a model of endothelial cells to investigate GDF15 expression during cellular senescence. Then, we analyzed the potential role of GDF15 in AB-ECFC functions and senescence. When AB-ECFCs become senescent, they secrete increased levels of GDF15. We investigated GDF15 paracrine effects on non-senescent AB-ECFCs and showed that GDF15 enhanced proliferation, migration, NO production and activated several signaling pathways including AKT, ERK1/2 and SMAD2 without triggering any oxidative stress. Taken together, our results suggest that GDF15 production by senescent AB-ECFCs could act in a paracrine manner on non-senescent AB-ECFCs, and that this interaction could be beneficial to its model cells. Therefore, GDF15 could play a beneficial role in a dysfunctional vascular system as previously reported in patients with CVD, by limiting ED related to vascular stress occurring in these diseases.

## Introduction

According to the World Health Organization, cardiovascular diseases (CVD) such as ischemic heart, cerebrovascular and peripheral arterial diseases have been the worldwide leading cause of death over the past 15 years. In vessels, the endothelium is composed of a monolayer of endothelial cells (ECs) involved in several physiological processes, including the control of vasomotor tone and the regulation of coagulation and inflammation [1,2]. However, with aging, the endothelium becomes progressively dysfunctional and its ability to maintain its full functions declines while emergence of CVD is observed [3].

Decrease nitric oxide (NO) bioavailability due to an increase in reactive oxygen species (ROS) [4] and a decrease in Nitric Oxide Synthase 3 (NOS3) activity [5] leads to impaired endothelial functions, including reduced vasomotor responses and increased levels of inflammatory and adhesion molecules [6] involved in CVD development.

Growth Differentiation Factor 15 (GDF15) is a stress response cytokine belonging to the Transforming Growth Factor β superfamily [7,8]. Physiologically, GDF15 is expressed at basal level in healthy human tissues except in the placenta, and is upregulated in several pathologies such as cancers [9–12], mitochondrial disorders [13] and CVD [14]. Among CVD, high GDF-15 levels have been found in peripheral artery diseases [15,16]. In addition, GDF15 has been described as a protective cytokine, preventing cold ischemia-reperfusion injury in heart transplantation [17] and limiting atherosclerosis development [18]. Furthermore, GDF15 is induced in several EC types in culture following a cellular stress such as a high glucose stimulus [19], oxygen concentration changes [20,21] or after chemotherapy treatment [9]. Taken together, these data suggest that GDF15 could play a role in endothelial damage and dysfunction. However, the functional relationship between GDF15 and EC physiology remains elusive because its biological effects vary according to EC type, environment and concentration [20,22,23].

Endothelial progenitor cells (EPCs) were discovered in 1997 [24] and are involved in endothelium homeostasis, through their physiological ability to regenerate the vascular system [25,26]. Endothelial Colony Forming Cells (ECFCs) are derived from EPCs in culture. They can be either isolated from peripheral adult blood (AB-ECFCs) or from umbilical cord blood (CB-ECFCs) [27]. CB-ECFCs exhibit high clonal, proliferative and functional capacities whereas AB-ECFCs rapidly undergo premature replicative senescence and display poor angiogenic potential [27–29]. Therefore, AB-ECFCs provide a convenient model to study EC senescence.

In this study, we hypothesized that GDF15 could be produced by ECs during a vascular stress to attenuate EC loss of function. Because cellular senescence is involved in vascular damage and stress, we used AB-ECFCs as a model of ECs to investigate GDF15 expression during senescence. Then, we investigated GDF15 biological effects in AB-ECFCs.

We first assessed the expression of GDF15 in ECs with different functional capacities. GDF15 was expressed both in AB-ECFCs and CB-ECFCs *in vitro* and a higher level of GDF15 was found in AB-ECFCs as compared to CB-ECFCs. This higher level of GDF15 was related to AB-ECFC senescence, while these cells became dysfunctional (decrease in their proliferation rate, angiogenic properties and NO bioavailability). Since circulating GDF15 was elevated during EC senescence, we then analyzed whether the addition of recombinant GDF15 to AB-ECFCs impacted their function. We showed that GDF15 improves AB-ECFC proliferation, migration, NO production and activated the AKT, ERK1/2 and SMAD2 signaling pathways without triggering oxidative stress. Taken together, our data suggest that in an impaired vascular system, dysfunctional ECs contribute to GDF15 production that could be beneficial to still functional endothelial cells as a tentative to delay or reduce endothelium damage.

## Materials and methods

Recombinant human GDF15 protein was purchased from Peprotech. GDF15 optimal concentration was determined by proliferation assay (S1 Fig) according to data from the literature (tested concentration range: 10–100 ng/mL). In this study, a GDF15 concentration of 50 ng/mL was found optimal. TGF-β, IL-6 and IL-8 ELISA kits were purchased from Abcam. Because it was described that recombinant GDF15 protein may contain traces of TGF-β contamination [30], we performed a TGF-β ELISA assay in EGM2 containing GDF15 recombinant protein at 50 ng/ml, the medium we used for our experiments and we did not revealed any detectable TGF-β contamination (S2 Fig).

### Blood source

Human sample collection and handling met the tenets of the declaration of Helsinki. Umbilical cord blood samples from healthy full-term newborns were obtained through a partnership with the Cord Blood Bank of St Louis Hospital (Paris, France), which is authorized by the French Regulatory Authority (authorization N° PPC51). Peripheral adult blood was obtained from human healthy males aged 18–70 years, through a partnership with the French Establishment of Blood (EFS, Ile de France, authorization 14/5/011). This activity was declared to and authorized by the French Ministry of Research under number AC-2008-376, and the French Organization for standardization under number 201/51848.1.

### ECFC isolation

ECFCs were isolated as previously described [29]. Briefly, mononuclear cells (MNC) from adult or cord blood were isolated using a Ficoll gradient. MNC were plated into collagen I (50 μg/mL) precoated 6- or 12-well plates in EGM2 medium (Lonza). The next day, cells were washed with 1X PBS and non-adherent cells were discarded daily for 7 days when the medium was changed. Thereafter, the medium was changed every other day. All cells were characterized by FACS using CD31, CD144 and KDR markers (S3 Fig).

### Antibodies

Rabbit anti-human antibodies against GDF15, peroxyredoxin-3 and peroxyredoxin-SO3 were purchased from Abcam. Anti-GFRAL antibody was purchased from Thermo Fisher Scientific. Anti-P44/42 anti-MAPK, anti-phospho-p44/42 MAPK, anti-Akt, anti-phospho-Akt, Smad2, and anti-phospho-Smad2 antibodies were purchased from Cell Signaling Technology.

### Western-blots

For classical western-blots, proteins were extracted using RIPA lysis buffer, then treated with dithiothreitol (100 mM final) and Laemmli (1X final). For redox western-blots, cells were treated with 20% trichloroacetic acid for 1h. Pellets were recovered and washed 3 times with cold acetone then dried. Dried pellets were resuspended in 70 μL of TES buffer (100 mM Tris-HCl, 1% SDS, 10 mM EDTA, pH 8.8) containing 100 mM N-Ethylmaleimide. Protein concentration was determined using Pierce BCA Protein Assay Kit. For redox western-blots, the lysates were subjected to SDS-PAGE under non-reducing conditions. Transfer was done onto PVDF membranes. After blocking, membranes were incubated with primary antibodies, then HRP-conjugated secondary antibody (Jackson Immunoresearch) and revealed with HRP substrate (Immobilon Western Chemiluminescent HRP Substrate) using enhanced chemiluminescence detection system (Fusion solo, Vilber lourmat).

### Measuring GDF15 secretion by AB-ECFC under a laminar flow

100.000 cells were seeded into a micro slide (Ibidi) in EGM2 for 2 hours then a 10μl/min flow was applied on these cells with a pressure pump (Fluigent, MFCS-EZ) for 48h. Then, supernatant was recovered and a GDF15 ELISA (Abcam) assay was performed following the manufacturer’s instructions.

### Senescence-associated β-galactosidase activity assay

Cells were seeded in triplicate into 12-well plates at a density of 5,000 cells/cm^2^. At 80% of confluence, a senescence-associated β-galactosidase activity assay was performed according to the manufacturer’s instructions (Sigma). After an overnight incubation with X-gal, cells were washed and five pictures (Nikon D5300) were taken per well at x10 magnification. Cells with positive and negative β-gal staining were counted using Image J cell counter software.

### ROS assessment using fluorescent probes

Cells were washed with PBS at 37°C and 1 mL of DMEM without phenol red (Thermo Fisher Scientific) containing 10 μM carboxy-H2DCFDA (Molecular Probes) or 5 μM MitoSOX (Molecular Probes) was added to each well. After a 40-min incubation at 37°C with 5% CO_2_, cells were collected, washed and resuspended in 500 μL of DMEM without phenol red. Green (530 nm) and red (585 nm) fluorescence was detected by flow cytometry using Accuri C6 cytometer (BD Biosciences) and mean fluorescence intensity (MFI) were quantified.

### RNA extraction, reverse transcription and quantitative RT-PCR

RNA extraction, reverse transcription and quantitative RT-PCR were performed as described previously [29]. Total RNA was extracted with RNeasy mini kit (Qiagen) and reverse transcription was performed with High-Capacity cDNA Reverse Transcription Kit (Thermo Fisher Scientific) following the manufacturer’s instruction. Quantitative Taqman RT-PCR was performed in triplicate with 7000 Real-time PCR system (Applied Biosystems, Themo Fisher Scientific). GDF-15: Hs00171132_m1, NQO1: Hs01045993_g1, GCLM: Hs00978072_m1 and HMOX1: Hs01110250_m1 probes were purchased from Applied Biosystems. The 2^ΔΔCt^ method was used to quantify the relative transcriptional level of each gene. Endogenous GAPDH was used as housekeeping gene.

### Cell viability

Cells were seeded in triplicate into a 96-well plate (5,000 cells/well) and incubated with EBM2 + 0.5% FBS for 7h. Then, cells were treated with 50 ng/mL of GDF15 overnight. Cell viability was assessed using the Cell titer gloR Luminescent cell viability assay (Promega) following the manufacturer’s instructions. Luminescence was measured using a Microplate Luminometer Centro LB 960 (Berthold Technologies).

### Cell cycle analysis

Cells were starved in EBM2 containing 0.5% FBS for 8h and then treated overnight with GDF15 (50 ng/mL). Cells were then detached and fixed with 70% cold ethanol for 2h. Then, cells were recovered, washed and resuspended in 1X PBS and incubated with 0.5 mg/mL of RNAse A for 1h at 37°C. Finally, cells were treated with 10 μg/mL of propidium iodide and analyzed by flow cytometry.

### BrDU incorporation assay

BrdU incorporation experiment was performed using the BrdU Cell Proliferation Assay Kit (Cell Signaling Technology) following the manufacturer’s instructions. Briefly, cells were seeded in triplicate into 96-well plates. The day after, cells were treated with EBM2 + 0.5% FBS for 8h. Then, cells were treated with GDF15 (50 ng/mL) or VEGF (50 ng/mL) in EBM2 + 0.5% FBS for 16h. BrdU was added for 4h in EGM2 without VEGF and FGF. Proliferation was quantified by measuring BrDU absorbance using a plate reader (Multiskan EX, Thermo Fisher Scientific).

### Cell migration assay

Cells were starved for 16h in EBM2 + 0.2% FBS and seeded into the upper compartment of fibronectin (bovine plasma) precoated (20 μg/mL, Sigma-Aldrich) 24-well, 8-μm culture inserts with transparent polyethylene terephthalate track-etched membrane (BD falcon). Then, GDF15 (50 ng/mL) and VEGF (50 ng/mL) were added into the lower compartment. After 5 hours, inserts were recovered, fixed with 4% paraformaldehyde and stained with May-Grünwald Giemsa coloration variant (RAL 555kit, RAL diagnostics, France). After cleaning the insert upper compartment with a cotton bud, we extracted and mounted the membrane from the insert. Membranes were photographed using a camera associated with an inverted microscope with a 10X objective in phase contrast mode. The number of cells was counted manually using Image J cell counter software.

### Assessment of nitric oxide production

DAF-FM diacetate (D23844, Invitrogen) was used to quantify NO production. At 80% confluence, cells were treated for 16h with GDF15 (50 ng/mL) or LPS (100 ng/mL) then washed and incubated with 1 μM DAF-FM diacetate probe in EBM2 + 0.5% FBS for 1h at 37°C. After removing the supernatant, cells were incubated in fresh EGM2 medium for 5 min at 37°C, resuspended in 1X PBS + 0.5% BSA and fluorescence intensity at 530 nm was quantified using Accuri C6 cytometer (BD Biosciences).

### Statistical analysis

All data were analyzed using Graph Pad 7.0 software. A Mann-Whitney T-test or Wilcoxon matched pairs signed rank t-test was used to compare two groups. Then, a Kruskal Wallis test was used. A non-parametric One-Way Anova test was used to compare three groups or more. Then, a Kruskal Wallis test and a Dunn’s multiple comparisons test was performed. A p value <0.05 was considered significant.

## Results

### GDF15 expression level is higher in AB-ECFCs than in CB-ECFCs

**Fig 1:**
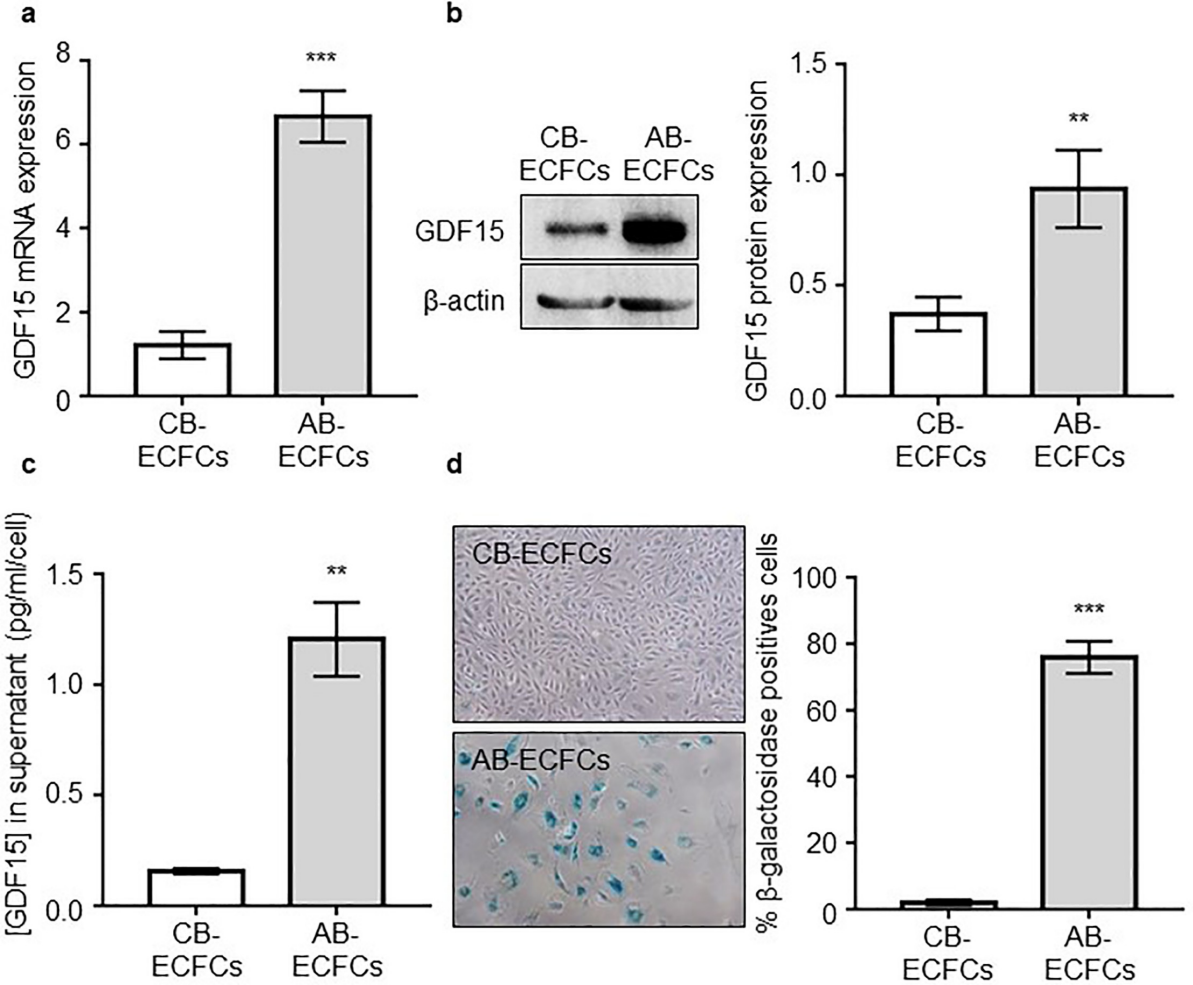
GDF15 level is increased in AB-ECFCs and appears to be related to senescence. Analysis of GDF15 expression and production by AB-ECFCs and CB-ECFCs between passages 4–5 by (**a**) RTqPCR (n = 7), (**b**) western-blot (n = 6) and (**c**) ELISA (n = 6). (**d**) Pictures and quantification of senescence by SA-β-gal assay in AB-ECFCs and CB-ECFCs (n = 7). Data are presented as the mean ± SEM. ** P <0.01 and *** P <0.001 compared to CB-ECFCs.

To evaluate GDF15 expression in and secretion AB-ECFCs and CB-ECFCs, we analyzed GDF15 gene and protein expression levels by RT-qPCR, Western-Blot and ELISA in these cells at same passages (between 4 and 5). We found that the levels of GDF15 mRNA (Fig 1A), and intracellular (Fig 1B) and extracellular (Fig 1C) proteins were significantly higher in AB-ECFCs than in CB-ECFCs (6.6, 2.5 and 8-fold higher, respectively). Using a Senescence Associated β-galactosidase assay (SA-β-gal), we showed a higher percentage of senescent cells in AB-ECFCs (75.96 ± 4.84%) than in CB-ECFCs (1.97 ± 0.79%, p = 0.0006, n = 7) (Fig 1D). These data suggested that ECs produce higher levels of GDF15 while they become senescent and dysfunctional.

### GDF15 expression and secretion are associated with senescence

**Fig 2:**
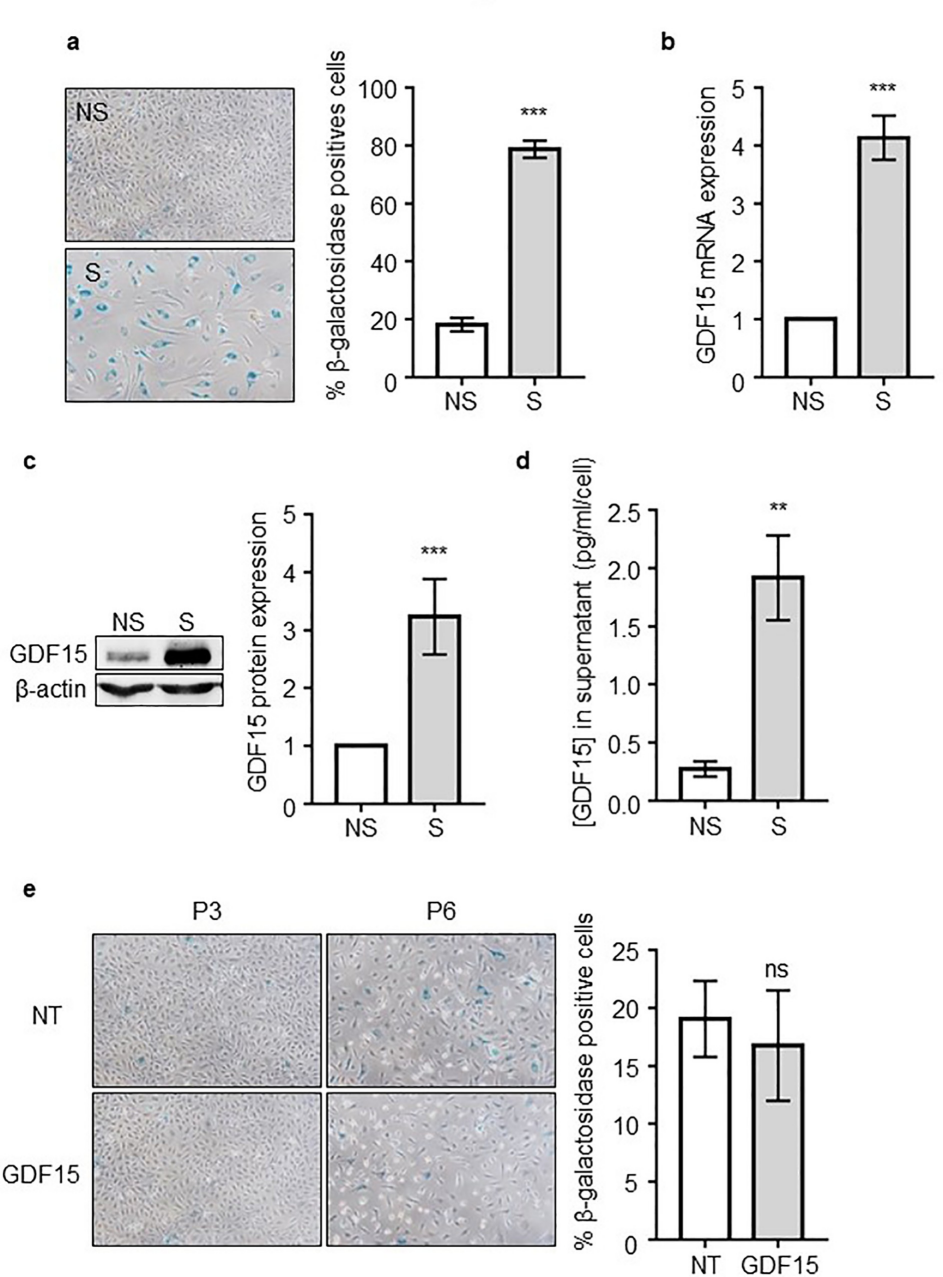
GDF15 expression and secretion is associated with senescence. (**a**) Pictures and quantification of NS AB-ECFCs and S AB-ECFCs stained using the SA-β-gal assay (n = 13). Analysis of GDF15 expression and secretion by NS AB-ECFCs and S AB-ECFCs by (**b**) RTqPCR (n = 13), (**c**) western-blot (n = 11) and (**d**) ELISA (n = 8). (**e**) GDF15 effects on cellular senescence from passages 3 to 6 (n = 3). Data are presented as the mean ± SEM. ns = non-significant, ** P <0.01 and *** P <0.001 compared to NS AB-ECFCs.

To confirm the relationship between GDF15 expression and AB-ECFC senescence, we measured GDF15 level in non-senescent (NS) and senescent (S) AB-ECFCs (Fig 2A). We found that GDF15 expression was strongly increased in S AB-ECFCs compared to NS AB-ECFCs by RTq-PCR (~ 4 folds) (Fig 2B), Western-Blot (~ 3.2 folds) (Fig 2C) and ELISA (~ 7 folds) (Fig 2D). In addition, we found that GDF15 expression was gradually increased along the successive passages (WB and ELISA) (S4 Fig) and we found that GDF15 was only over-secreted by senescent AB-ECFCs under a laminar flow condition (S5 Fig). Moreover, GDF15 overexpression was also found in senescent CB-ECFCs, which senesce very lately beyond passage 20 (S6 Fig). These results confirm that GDF15 expression is related to AB-ECFCs senescence. Beside GDF15 overexpression in senescent AB-ECFCs, we also found that IL-6 and IL-8 pro-inflammatory cytokines, hallmark of senescent cells [31], were also over-secreted by these cells (S7 Fig). To identify the role of GDF15 in senescence induction, we added a recombinant human GDF15 protein at 50 ng/mL to AB-ECFC cultures at each passage from passages 3 to 6. After these three passages, we observed that the number of β-galactosidase positive cells was not increased in GDF15-treated cultures (Fig 2E). Thus, recombinant GDF15 had no significant effect on senescence induction in AB-ECFCs.

These data confirmed that GDF15 upregulation and expression is significantly associated with AB-ECFC senescence and that this expression does not increase the senescence progression rate.

### GDF15 improves AB-ECFC functions

**Fig 3:**
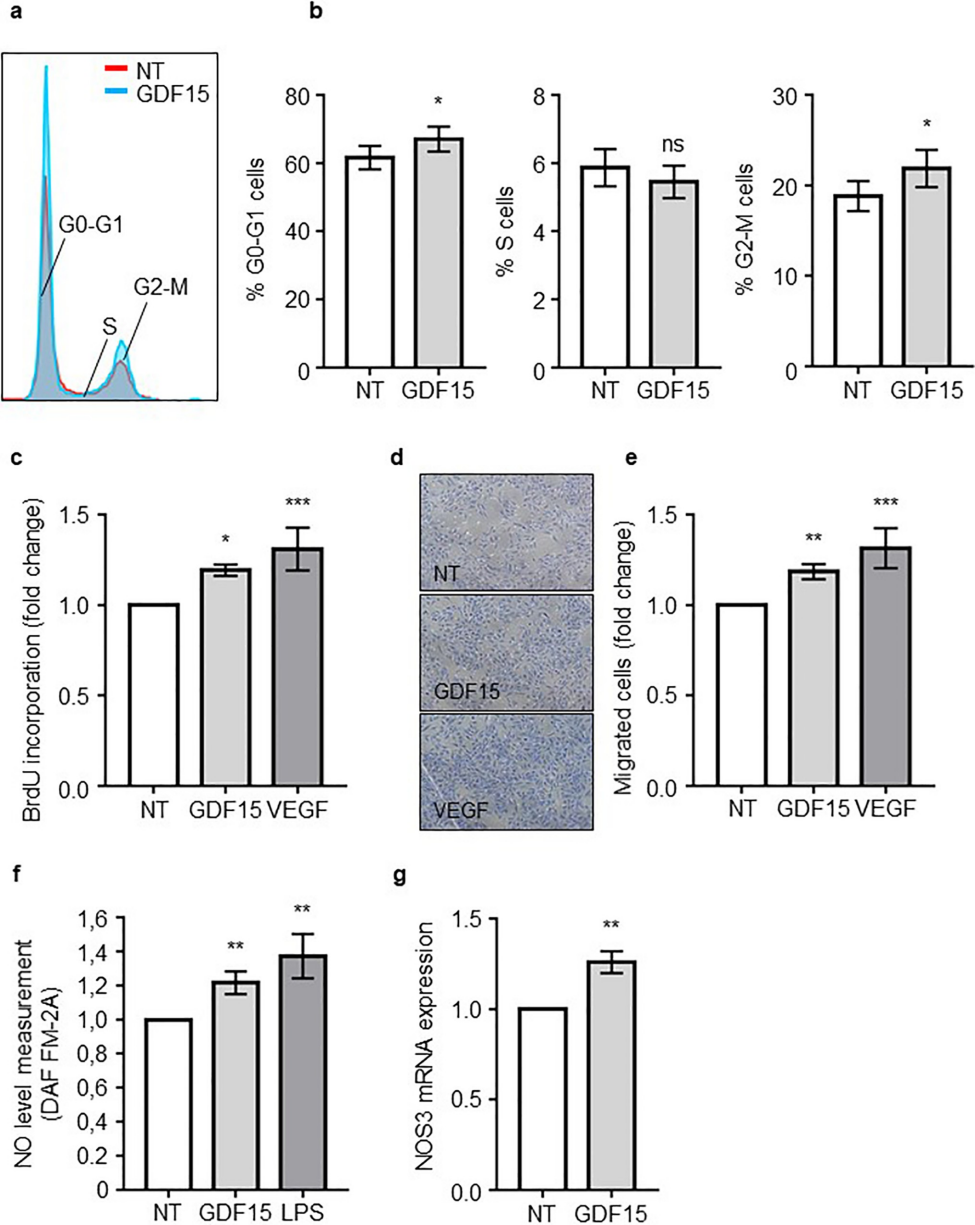
GDF15 improves AB-ECFC functions. AB-ECFCs were starved for 8h and treated overnight with GDF15 (50 ng/mL) or VEGF (50 ng/mL), then (**a**) the cell cycle was analyzed by FACS focusing on the G0-G1, S and G2-M phases (n = 6). Quantifications are presented in (**b**). BrdU proliferation assay was performed on AB ECFCs in the presence of GDF15 or VEGF (**c**). Migration assays were performed after GDF15 (50 ng/mL) or VEGF (50 ng/mL) treatment. Representative pictures and quantification of AB-ECFC migration assays are shown in (**d**) and (**e**), respectively (n = 4). (**f**) Intracellular nitric oxide (NO) MFI analyzed by flow cytometry using DAF-FM-2A probes in NT, LPS- (100 ng/mL) and GDF15- (50 ng/mL) treated AB-ECFCs (n = 8). (**g**) *NOS3* RTqPCR analysis in NT and GDF15-treated AB-ECFCs (n = 8). Data are presented as the mean ± SEM. ns = non-significant, * P <0.05, ** P <0.01 and *** P <0.001 compared to NT AB-ECFCs.

Since S AB-ECFCs secreted high levels of GDF15, we then investigated the paracrine effect of GDF15 on NS AB-ECFCs. We added recombinant GDF15 to NS AB-ECFC cultures and analyzed its effects on cell proliferation, migration and NO production. We first investigated whether GDF15 treatment modified cell cycle progression in AB-ECFC cultures. GDF15-treated and non-treated (NT) cells were labeled with propidium iodide and their cell cycle profile was analyzed by flow cytometry. Here, VEGF at 50 ng/mL was used as a positive control. In the presence of GDF15, the number of cells in G0-G1 (61.6 ± 3.4% in NT cells and 67.1 ± 3.6% in GDF15-treated cells) and in G2-M (18.8 ± 1.6% in NT cells and 21.9 ± 2% in GDF15-treated cells) phases was increased (Fig 3A and 3B). Then, we showed a 20% increase in BrDU incorporation in GDF15-treated cells compared to NT cells (p <0.0001, n = 6) (Fig 3C). We then investigated the effect of GDF15 on AB-ECFC migration capabilities using a transwell migration assay. GDF15 significantly improved AB-ECFC migration compared to NT cells (p = 0.0012, n = 4), reaching a migration level similar to that obtained with VEGF (Fig 3D and 3E). Then, we used a specific probe (DAF-FM-2A) to quantify intracellular NO in AB-ECFCs. Here, LPS was used as positive controls. GDF15- and LPS-treated cells contained a higher level of NO (~21% and ~37%, respectively) compared to NT cells (p = 0.0021, n = 8) (Fig 3F, data were normalized to MFI of NT cells). Simultaneously, we analyzed *NOS3* expression by RT-PCR in AB-ECFCs treated or not with GDF15. NOS3 is the endothelium-specific nitric oxide synthase [32]. *NOS3* mRNA was upregulated in GDF15-treated cells (~26%) compared to NT cells (Fig 3G). Taken together, these results demonstrated that GDF15 improves AB-ECFC proliferation, migration and induction of NO production.

### GDF15 induces the production of non-deleterious superoxide ions

**Fig 4:**
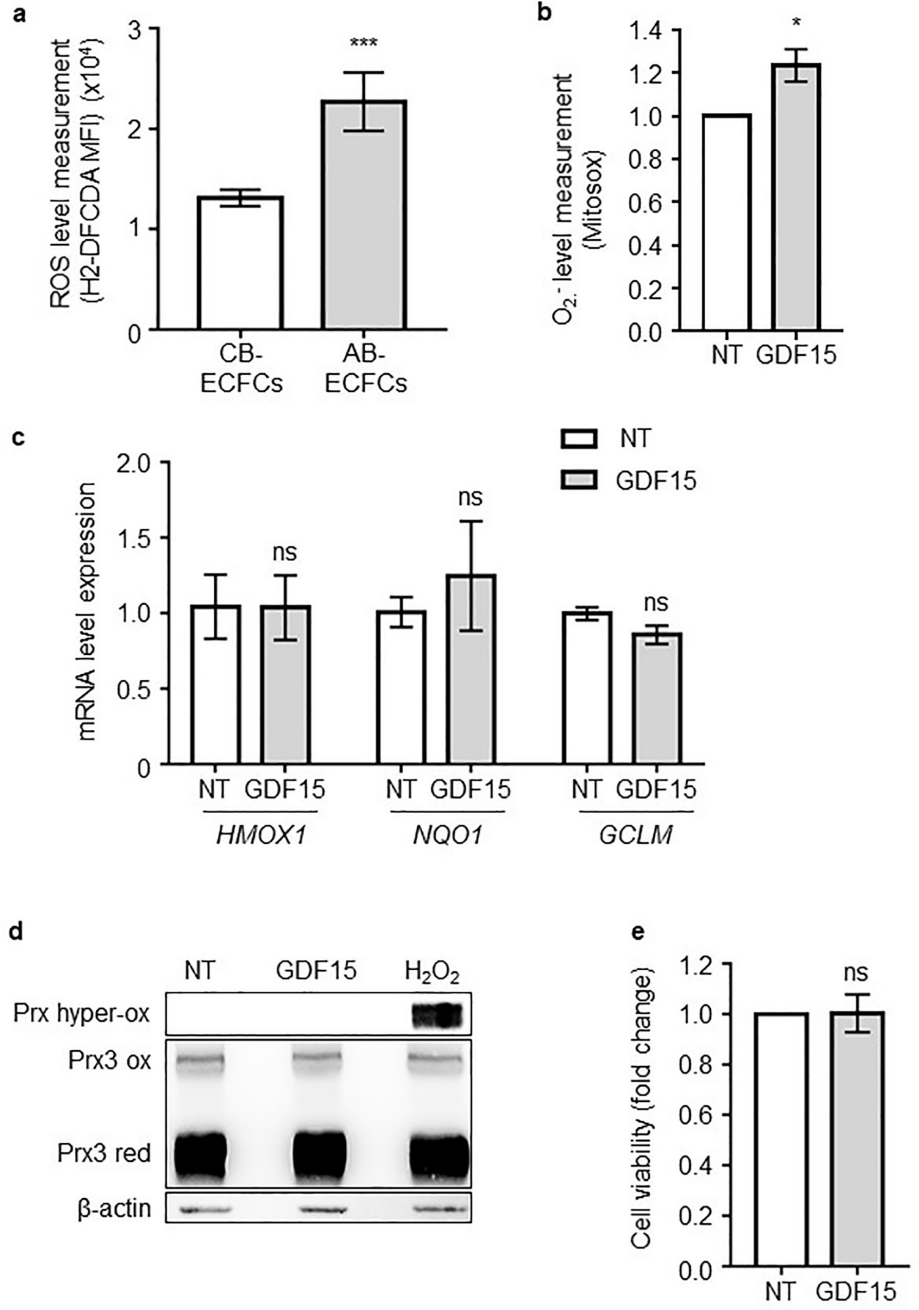
GDF15 induces a non-deleterious superoxide ion production. (**a**) Intracellular ROS level measurement using H2-DCFDA probes in CB-ECFCs (n = 8) and AB-ECFCs (n = 6). (**b**) Mitochondrial superoxide ion quantification using Mitosox probes in NT or GDF15- (50 ng/mL) treated AB-ECFCs (n = 5). MFI detected for each probe were measured by FACS. (**c**) Analysis of *HMOX1*, *GCLM* and *NQO1* expression by RT-qPCR (n = 3). (**d**) Redox western-blot of peroxiredoxin 3 (Prx3) protein in NT, GDF15- (50 ng/mL) or H2O2- (400 μM) treated AB-ECFCs (n = 3). (Prx3 red: Peroxireoxin 3 reduced form, Prx3 ox: Peroxiredoxin 3 oxidized form, Prx hyper-ox: Peroxiredoxin hyper-oxidized form). β-actin was used as the loading control. (**e**) Cell viability analysis in NT and GDF15-treated AB-ECFCs (n = 5). Data are presented as the mean ± SEM. ns = non-significant and * P <0.05, ** P <0.01 and *** P <0.001 compared to CB-ECFCs or NT cells.

To compare basal ROS levels between AB-ECFCs and CB-ECFCs, H2-DCFDA, a specific intracellular probe whose oxidization is proportional to the amount of ROS present in the cells, was used. Quantification of H2-DCFDA MFI by flow cytometry showed that the basal ROS level was ~2 times higher in AB-ECFCs than in CB-ECFCs (p = 0.0021, n = 5) (Fig 4A).

As ROS are byproducts of cellular proliferation and migration [33] which are rapidly generated by mitochondria, we then investigated after only 1h of GDF15 treatment on AB-ECFCs whether superoxide ions, the main mitochondrial ROS byproduct [34], were produced. The analysis of Mitosox, a mitochondrial superoxide probe, revealed that GDF15 treatment led to a 21% increase in superoxide production compared to NT cells (Fig 4B). However, the basal ROS level was already high in AB-ECFCs. We then investigated whether superoxide ion production could be toxic to AB-ECFCs. NRF2 is the main transcription factor responsible for the induction of key antioxidant enzymes under oxidative stress conditions [35]. We did not detect any significant increase in mRNA levels of 3 genes regulated by NRF2 (*GCLM*, *HMOX1* and *NQO1*) in AB-ECFCs after GDF15 treatment (Fig 4C). This showed that the GDF15-induced increase in superoxide ion production in AB-ECFCs did not trigger any oxidative stress. Peroxiredoxin 3 (PRX3) is the main mitochondrial peroxiredoxin to be readily oxidized by hydrogen peroxide (H_2_O_2_), a ROS produced after superoxide dismutation. The evaluation of the PRX3 redox status after treatment of AB-ECFCs with GDF15 or H_2_O_2_ (400 μM) revealed that, unlike H_2_O_2_, GDF15 did not lead to the accumulation of the hyper-oxidized form of PRX3 (Fig 4D). This suggested that the superoxide ions produced following GDF15 treatment were properly scavenged by the antioxidant system. Furthermore, under these conditions, GDF15 was not toxic to AB-ECFCs as the cell viability remained unchanged after GDF15 treatment (Fig 4E), confirming the absence of oxidative stress despite superoxide ion production.

Taken together, these results suggested that treatment of AB-ECFCs with GDF15 generates superoxide ions but at a higher level than the threshold required for PRX3 oxidation and for NRF2 activation, in response to oxidative stress.

### GDF15 activates the ERK1/2, SMAD2 and AKT signaling pathways

**Fig 5:**
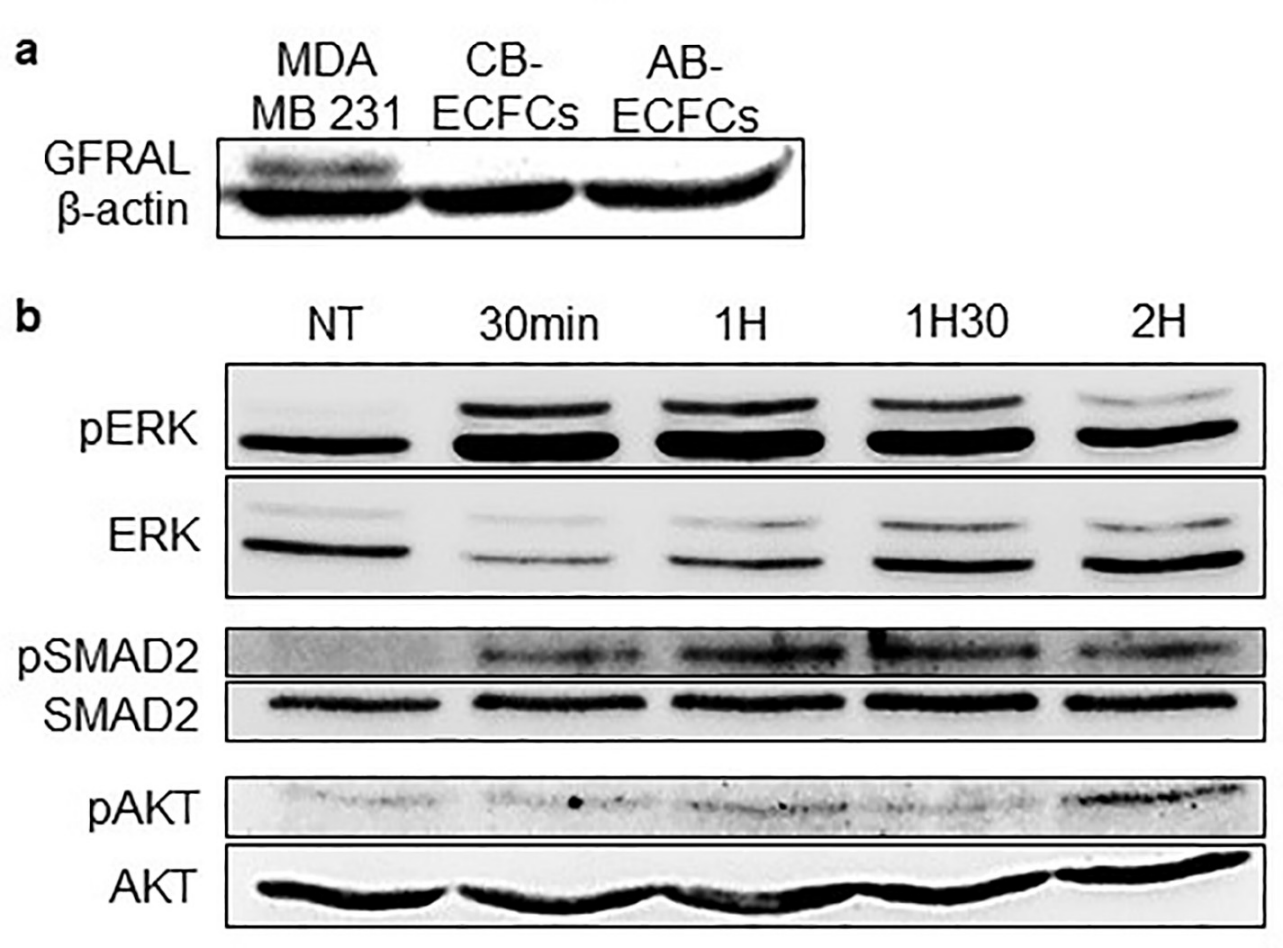
GDF15 activates ERK1/2, SMAD2 and AKT signaling pathways. (**a**) Western-blot of GFRAL in MDA-MB 231 cells (positive control), CB-ECFCs and AB-ECFCs. (**b**) Western-blot of pERK1/2, ERK1/2, pSMAD2, SMAD2, pAKT and AKT in AB-ECFCs treated with GDF15 (50 ng/mL) at different timepoints: 30 min, 1h, 1h30 and 2h.

We then analyzed whether the GDF15 receptor, GFRAL, the presence of which has recently been identified in the nervous system [36–39], was present on CB-ECFCs and AB-ECFCs and thus could also be the GDF15 receptor on ECs. The Western-Blot analysis did not reveal any expression of GFRAL in AB-ECFCs and CB-ECFCs, unlike in MDA MB231 cells that were used as a positive control (Fig 5A).

Then, we focused on several previously described signaling pathways known to be activated by GDF15 [19,22,40,41]. Western-Blots showed that GDF15 activated the ERK1/2, SMAD2 and AKT signaling pathways (Fig 5B).

Here, we showed that GFRAL is not the receptor of GDF15 on AB-ECFCs and CB-ECFCs and that GDF15 activates the ERK1/2, SMAD2 and AKT signaling pathways in AB-ECFCs.

## Discussion

Endothelial dysfunction plays an important role in CVD progression. GDF15, a circulating cytokine normally expressed at basal levels in most human tissues in steady-state situations, is overexpressed in various pathologies, including CVD [15–17,42], or in response to cellular stresses ^12–25^, in particular by ECs.

Based on these observations, we assumed that circulating GDF15 could be produced by ECs during vascular stress to attenuate EC loss of function and damage. In this study, we used AB-ECFCs and CB-ECFCs, which are the EPCs progeny obtained in culture, as models of ECs displaying different functional properties. Indeed, CB-ECFCs are highly active ECs and are resistant to replicative senescence whereas AB-ECFCs are less active and rapidly undergo replicative senescence.

We determined whether GDF15 was differentially expressed in these ECs with contrasted functionalities. GDF15 was specifically overexpressed in AB-ECFCs between passages 4 and 5 or when enforcing a laminar flux, while it was expressed in CB-ECFCs at lower levels. We also showed that AB-ECFCs included a higher proportion of senescent cells than CB-ECFCs at the same passage, thus suggesting a relationship between GDF15 expression and cellular senescence. These findings are consistent with our previous study [29] showing that the number of β-galactosidase positive cells (senescent) was higher in AB-ECFCs compared to CB-ECFCs.

Then, to determine whether GDF15 expression was associated to cellular senescence, it expression was quantified in AB-ECFCs and CB-ECFCs. We found that AB-ECFCs expressed basal levels of GDF15 at early passages when they are not yet senescent, like in CB-ECFCs. GDF15 level then raised progressively while the number of β-galactosidase positive cells increased. GDF15 expression was maximal when most AB-ECFCs were senescent. In other studies, GDF15 has also been shown to be overexpressed in radiation-induced senescent HAECs [40], supporting the relationship between senescence and GDF15 over expression. Taken together, all these findings suggest that senescent ECs could contribute to the release of high GDF15 levels as previously found in patients with CVD [14–16]. Therefore, GDF15 could be referred to as a “senescence-associated secreted protein” (SASP) like IL-6 or IL-8 which are also secreted by senescent AB-ECFCs.

Senescence may be induced by multiple cellular stresses such as telomere attrition, genomic damage and oncogenic stress [43]. One of the hallmarks of senescent cells is the increased expression and production of SASP, that include several types of factors such as proinflammatory cytokines, interleukins, chemokines, growth factors and proteases [31]. SASP contribute to create a proinflammatory environment that influences neighboring cell behavior [31,44–46]. In order to analyze the paracrine effect of GDF15 on ECs, we treated non senescent AB-ECFCs with recombinant GDF15 to determine whether this could affect their functionality. We first found that the long-term treatment of non senescent AB-ECFCs with recombinant GDF15 neither contributed nor delayed their senescence. Recently, GDF15 has been shown to improve HUVEC proliferation and angiogenic capacities [20,47]. These data are consistent with our current findings because we demonstrate that GDF15 improves AB-ECFC proliferation, migration and NO production. This suggests thus that high levels of GDF15 is beneficial for a dysfunctional endothelial system.

Mitochondria play a major role in cellular metabolism and are the main source of ROS [48]. ROS may trigger various cellular responses depending on their concentrations [33]. At high concentrations, ROS may trigger oxidative stress resulting in a stress adaptive response, cellular damages or apoptosis [33]. Beside GDF15 positive effects on ECs, GDF15 could also have toxic effects, since it induces the production of ROS, as shown in previous studies [19,40]. Since AB-ECFCs express higher ROS levels than CB-ECFCs, we showed that GDF15 treatment led to an additional mitochondrial superoxide ion (O₂⁻) production, a main ROS byproduct that could trigger a toxic oxidative stress [33]. In order to evaluate the possible deleterious effects of this O₂⁻ production, we analyzed different markers of cellular response to oxidative stress. First, we focused on *NRF2*, the main transcription factor regulating the anti-oxidant response. We showed that *NRF2-*dependent gene (*GCLM*, *NQO1* and *HMOX1*) expression was not induced by GDF15 treatment, indicating that the cellular response to oxidative stress was not activated. Second, we did not detect any peroxiredoxin hyper-oxidation, a phenomenon due to a mitochondrial anti-oxidant enzyme, indicating that the protein oxidation pathway was not induced. Third, GDF15 treatment did not affect AB-ECFC viability and senescence status. Taken together, in a context of high ROS levels, our results demonstrated that, despite an increase in O₂⁻ production, GDF15 treatment of NS AB-ECFCs has beneficial effects.

Finally, we investigated GDF15 signaling pathways. GFRAL, previously described as an orphan receptor expressed on area postrema neurons and solitary tract nuclei in mice and humans, has been recently identified as a specific receptor of GDF15 [36–39]. We assessed whether AB-ECFCs, that are sensitive to GDF15 actions, expressed GFRAL. We did not detect any GFRAL expression in AB-ECFCs and CB-ECFCs, suggesting that GFRAL is not the GDF15 receptor on ECFCs. Therefore, GDF15 might use another receptor that remains to be identified or a receptor shared with other TGF-β molecules, as previously suggested [49,50]. Moreover, we demonstrated that GDF15 activated several signaling pathways such as the ERK1/2, AKT and SMAD2 pathways. Several studies have described that the activation of these pathways induces EC proliferation, migration, NO production and angiogenesis [19,22,40,41], which is confirmed and supported by our findings. These results thus confirmed that GDF15 is able to activate various signaling pathways to achieve its functions.

In summary, we showed that GDF15, produced by senescent ECs, improves AB-ECFC proliferation, migration and NO production through the activation of various signaling pathways. We also confirmed that GDF15 induces ROS production and showed for the first time that GDF15 is not deleterious to ECs because it does not trigger any oxidative stress. Therefore, GDF15 could have a therapeutic effect on a damaged vascular system. However, further studies in animal models are needed to confirm GDF15 beneficial effects on the vascular system.

## Supporting information

S1 Fig GDF15 optimal concentration determined using a proliferation assay. BrdU incorporation assay in AB-ECFCs in the presence of 10, 50 and 100 ng/mL of GDF15.
(TIF)

S2 Fig TGF-β is not detected in GDF15 prep. ELISA of TGF-β protein in EGM2 or in EGM2 supplemented with 50ng/ml of GDF15. The point TGF-β at 32pg/ml of the ELISA curve is used here as positive control. ND: non detected
(TIF)

S3 Fig AB-ECFC and CB-ECFC phenotypic characterization. (**a**) Pictures and (**b**) phenotypic characterization by FACS of AB-ECFC and CB-ECFC colonies. AB-ECFCs and CB-ECFCs are both positive for the CD31, CD144 and KDR endothelial markers and negative for the CD45 hematopoietic marker.
(TIF)

S4 Fig GDF15 expression gradually increases with passages. GDF15 expression by (**a**) western-blot and (**b**) ELISA between passages 3 to 5.
(TIF)

S5 Fig GDF15 is over-secreted by senescent AB-ECFC under a laminar flow. ELISA of GDF15 in non senescent (NS) and senescent (S) AB-ECFC under a laminar flow (n = 6). Data are presented as the mean ± SEM. * P <0.05, compared to NS AB-ECFC.
(TIF)

S6 Fig GDF15 mRNA is also over-expressed in senescent CB-ECFC. RT-PCR analysis (n = 5) in senescent AB-ECFC (around passage 5), in CB-ECFC at passage 5, 10 and in senescent CB-ECFC (beyond passage 20). Data are presented as the mean ± SEM. ns = non-significant and * P <0.05, compared to AB-ECFCs.
(TIF)

S7 Fig IL-6 and IL-8 are over-secreted in senescent AB-ECFC. Analysis of (a) IL-6 (n = 8) and (b) IL-8 (n = 7) secretion in non senescent (NS) and senescent (S) AB-ECFC in ELISA. Data are presented as the mean ± SEM. ** P <0.01 compared to NS AB-ECFCs.
(TIF)
