# Supplementary figures and images for: GDF15 secreted by senescent endothelial cells improves vascular progenitor cell functions
Source: PLoS One. 2019 May 10;14(5):e0216602. doi: 10.1371/journal.pone.0216602 (PMC6510423; doi:10.1371/journal.pone.0216602)

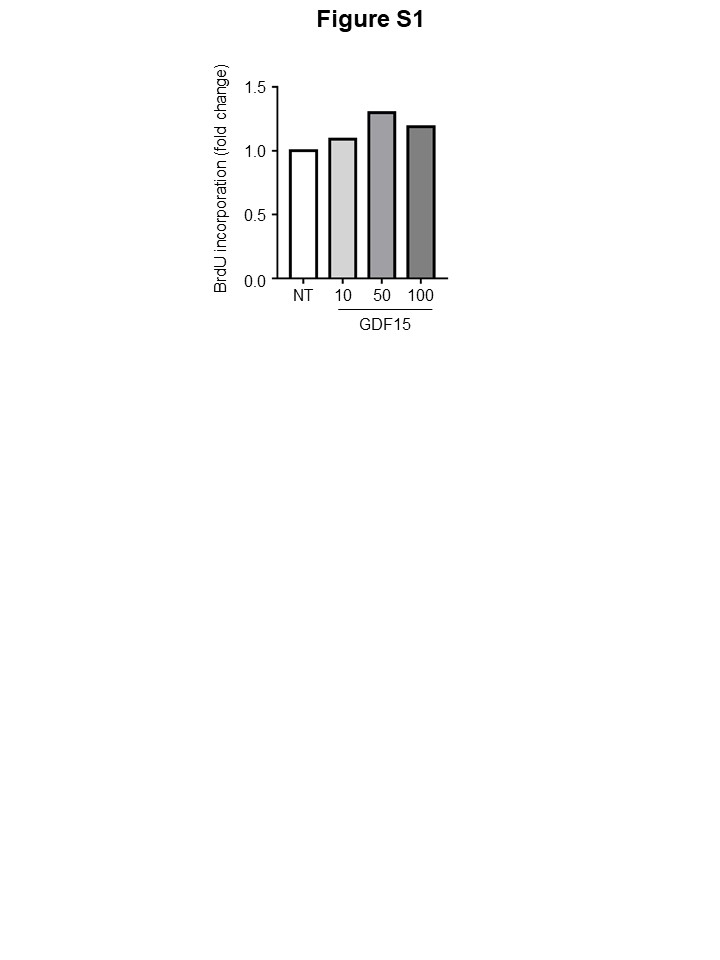

Supplement: S1 Fig — BrdU incorporation assay in AB-ECFCs in the presence of 10, 50 and 100 ng/mL of GDF15. (TIF) [file pone.0216602.s001.tif]

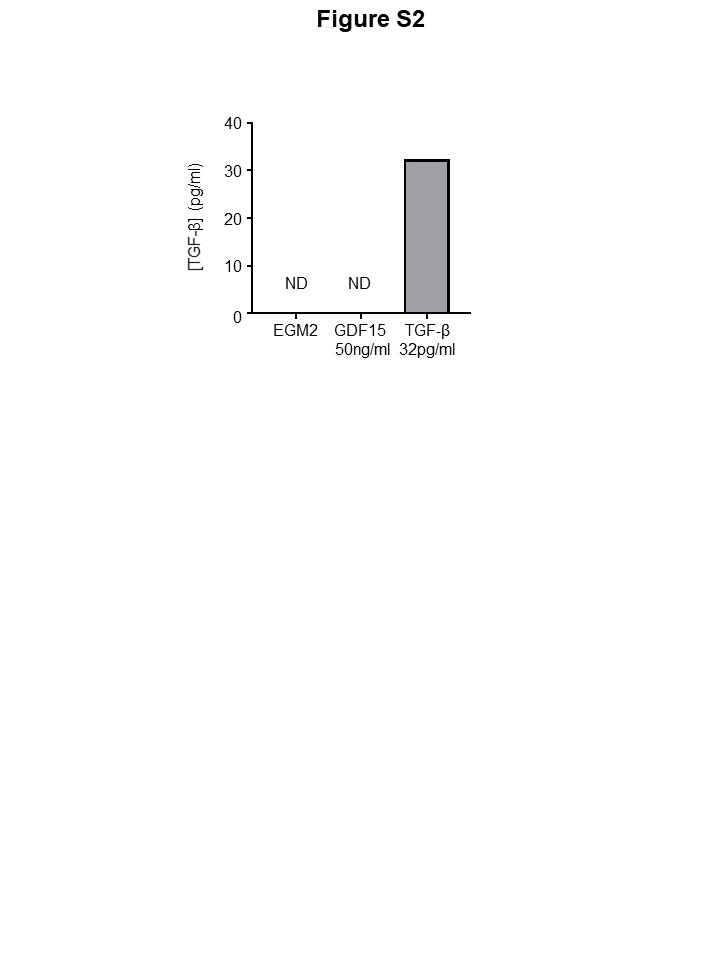

Supplement: S2 Fig — ELISA of TGF-β protein in EGM2 or in EGM2 supplemented with 50ng/ml of GDF15. The point TGF-β at 32pg/ml of the ELISA curve is used here as positive control. ND: non detected (TIF) [file pone.0216602.s002.tif]

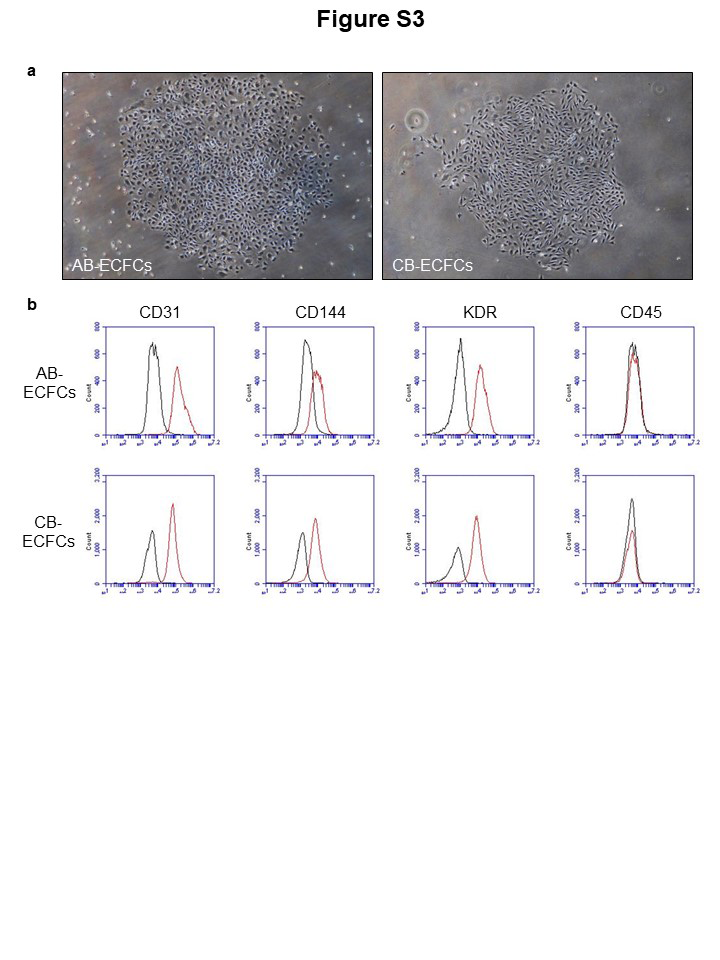

Supplement: S3 Fig — (a) Pictures and (b) phenotypic characterization by FACS of AB-ECFC and CB-ECFC colonies. AB-ECFCs and CB-ECFCs are both positive for the CD31, CD144 and KDR endothelial markers and negative for the CD45 hematopoietic marker. (TIF) [file pone.0216602.s003.tif]

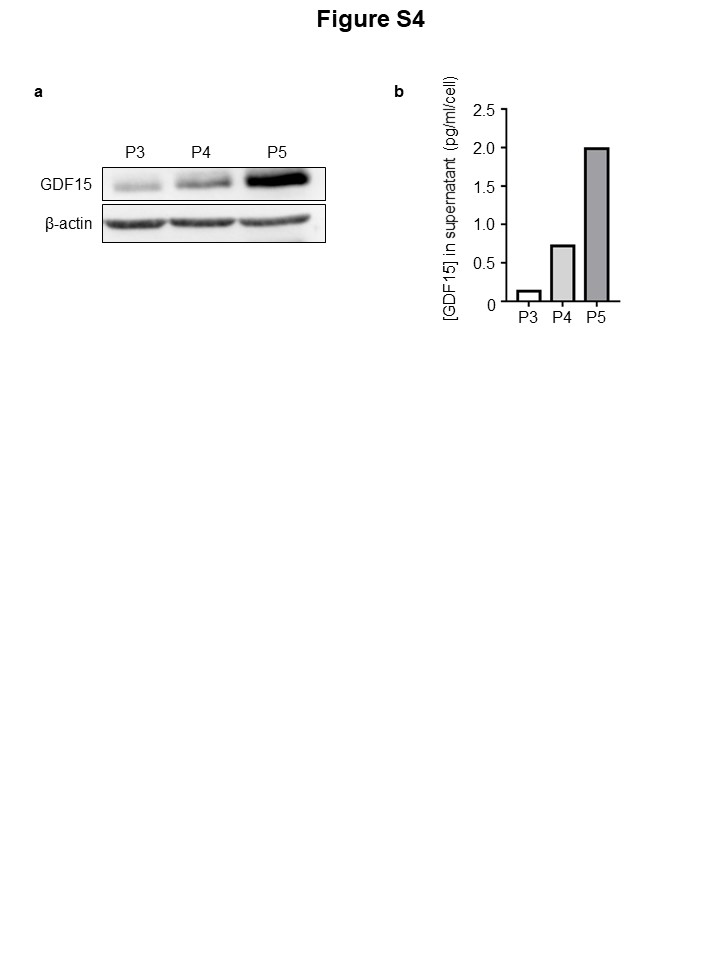

Supplement: S4 Fig — GDF15 expression by (a) western-blot and (b) ELISA between passages 3 to 5. (TIF) [file pone.0216602.s004.tif]

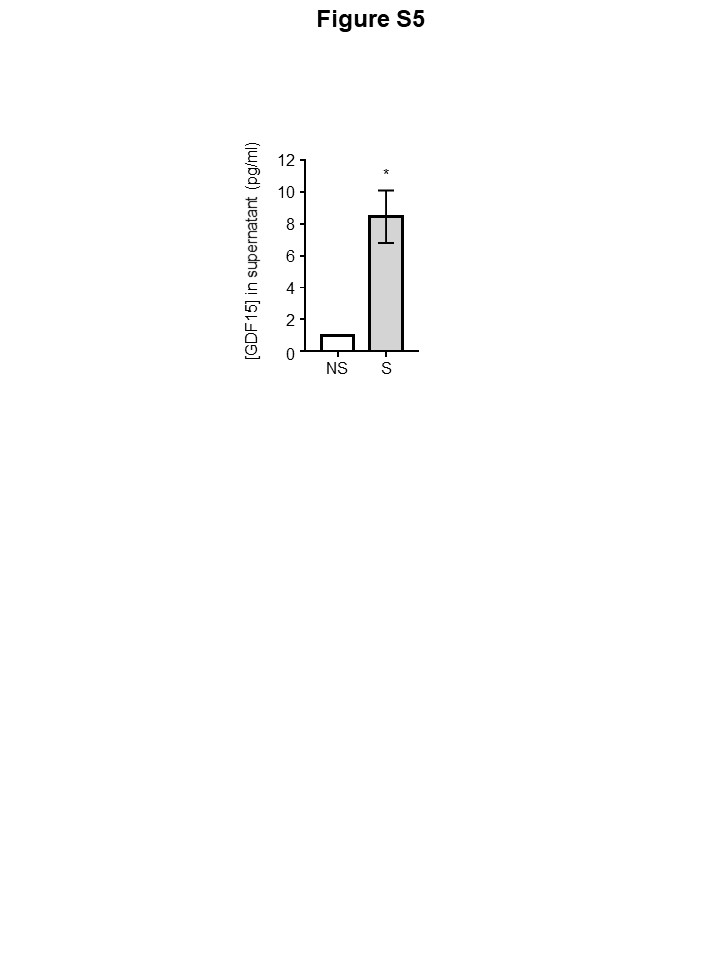

Supplement: S5 Fig — ELISA of GDF15 in non senescent (NS) and senescent (S) AB-ECFC under a laminar flow (n = 6). Data are presented as the mean ± SEM. * P <0.05, compared to NS AB-ECFC. (TIF) [file pone.0216602.s005.tif]

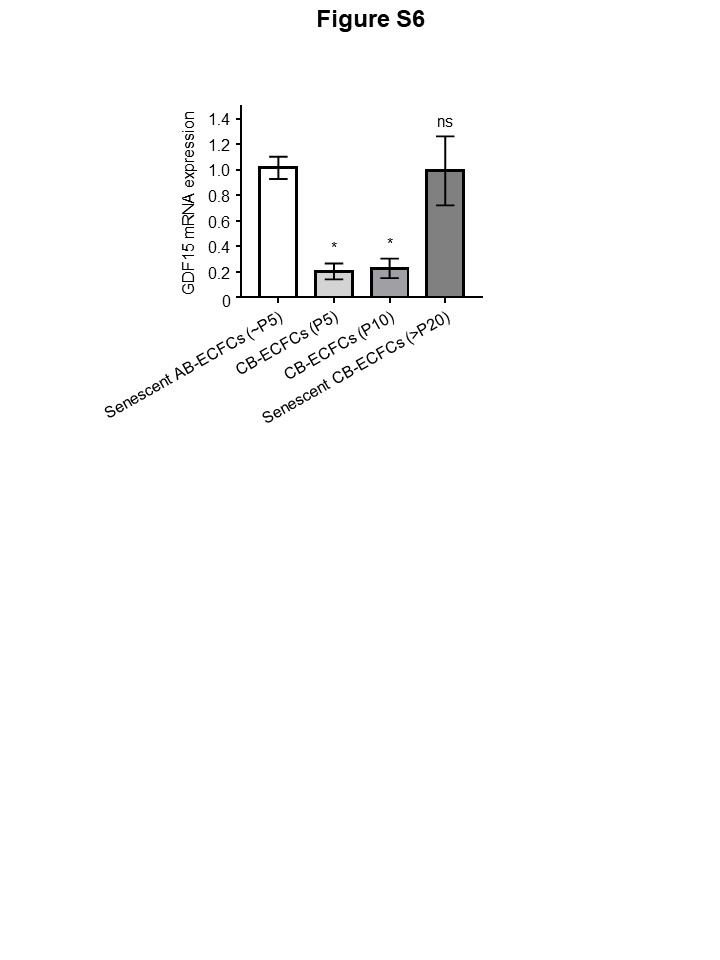

Supplement: S6 Fig — RT-PCR analysis (n = 5) in senescent AB-ECFC (around passage 5), in CB-ECFC at passage 5, 10 and in senescent CB-ECFC (beyond passage 20). Data are presented as the mean ± SEM. ns = non-significant and * P <0.05, compared to AB-ECFCs. (TIF) [file pone.0216602.s006.tif]

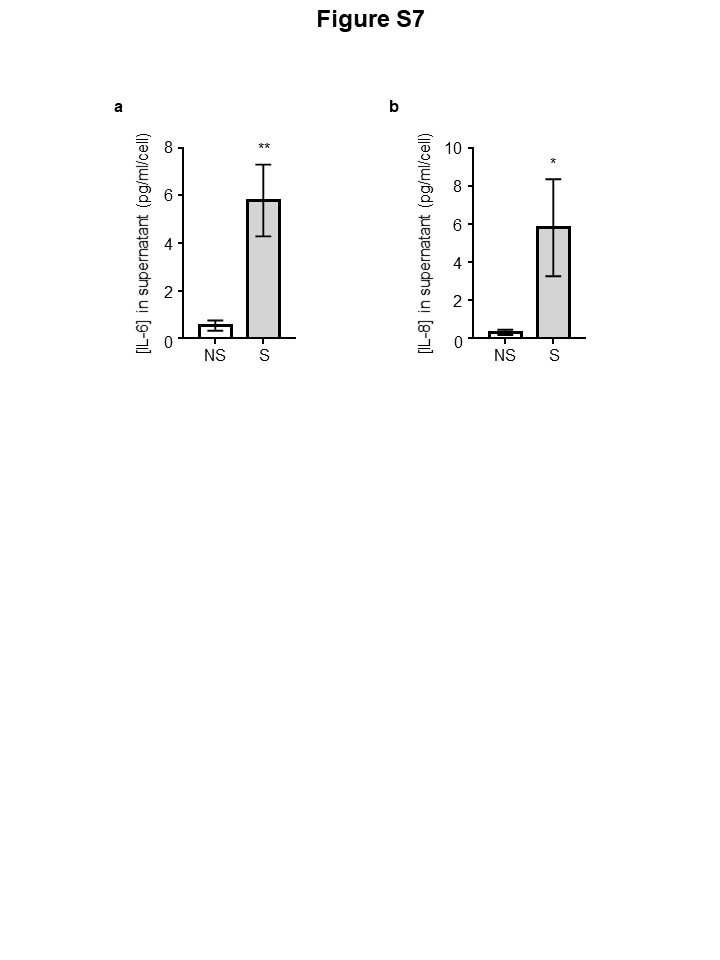

Supplement: S7 Fig — Analysis of (a) IL-6 (n = 8) and (b) IL-8 (n = 7) secretion in non senescent (NS) and senescent (S) AB-ECFC in ELISA. Data are presented as the mean ± SEM. ** P <0.01 compared to NS AB-ECFCs. (TIF) [file pone.0216602.s007.tif]
